# Novel Ca-Chelating Peptides from Protein Hydrolysate of Antarctic Krill (*Euphausia superba*): Preparation, Characterization, and Calcium Absorption Efficiency in Caco-2 Cell Monolayer Model

**DOI:** 10.3390/md21110579

**Published:** 2023-11-05

**Authors:** Ming-Xue Ge, Ru-Ping Chen, Lun Zhang, Yu-Mei Wang, Chang-Feng Chi, Bin Wang

**Affiliations:** 1 Zhejiang Provincial Engineering Technology Research Center of Marine Biomedical Products, School of Food and Pharmacy, Zhejiang Ocean University, Zhoushan 316022, China; gmx8670@163.com (M.-X.G.); 15157780517@163.com (R.-P.C.); 2 National and Provincial Joint Laboratory of Exploration and Utilization of Marine Aquatic Genetic Resources, National Engineering Research Center of Marine Facilities Aquaculture, School of Marine Science and Technology, Zhejiang Ocean University, Zhoushan 316022, China; zl15525864652@163.com (L.Z.);

**Keywords:** Antarctic krill (*Euphausia superba*), Ca-chelating peptide, VERG, property analysis, absorption efficiency, Caco-2 cell model

## Abstract

Antarctic krill (*Euphausia superba*) is the world’s largest resource of animal proteins and is thought to be a high-quality resource for future marine healthy foods and functional products. Therefore, Antarctic krill was degreased and separately hydrolyzed using flavourzyme, pepsin, papain, and alcalase. Protein hydrolysate (AKH) of Antarctic krill prepared by trypsin showed the highest Ca-chelating rate under the optimized chelating conditions: a pH of 8.0, reaction time of 50 min, temperature of 50 °C, and material/calcium ratio of 1:15. Subsequently, fourteen Ca-chelating peptides were isolated from APK by ultrafiltration and a series of chromatographic methods and identified as AK, EAR, AEA, VERG, VAS, GPK, SP, GPKG, APRGH, GVPG, LEPGP, LEKGA, FPPGR, and GEPG with molecular weights of 217.27, 374.40, 289.29, 459.50, 275.30, 300.36, 202.21, 357.41, 536.59, 328.37, 511.58, 516.60, 572.66, and 358.35 Da, respectively. Among fourteen Ca-chelating peptides, VERG presented the highest Ca-chelating ability. Ultraviolet spectrum (UV), Fourier Transform Infrared (FTIR), and scanning electron microscope (SEM) analysis indicated that the VERG-Ca chelate had a dense granular structure because the N-H, C=O and -COOH groups of VERG combined with Ca^2+^. Moreover, the VERG-Ca chelate is stable in gastrointestinal digestion and can significantly improve Ca transport in Caco-2 cell monolayer experiments, but phytate could significantly reduce the absorption of Ca derived from the VERG-Ca chelate. Therefore, Ca-chelating peptides from protein hydrolysate of Antarctic krill possess the potential to serve as a Ca supplement in developing healthy foods.

## 1. Introduction

Dietary minerals as micronutrients have an extremely crucial impact on human health, but some factors, such as diet, lifestyle, habits, living environment, and genetics, can make people subjected to mineral deficiency and lead to many types of illness [1]. For instance, zinc deficiency influences about 2 billion people and results in immune dysfunction, delayed wound healing, growth retardation, hypogonadism, hair loss, and brain atrophy and dysfunction; iron and copper deficiency may result in impaired hematopoiesis function, iron deficiency anemia, and neurological disorders [2]. The human body contains about 700–1400 g of calcium (Ca), which is the richest inorganic element and is involved in a variety of physiological activities, including bone strength, neurotransmission, blood coagulation, enzyme activation, cellular proliferation, and muscle contraction [3]. In addition, Ca also takes part in regulating neural activity and the permeability of cell membranes. Ca-deficiency can lead to osteoporosis, metabolic disorders, rickets, intestine cancer, osteomalacia, and hypertension [4,5]. Compared with the recommended daily allowance of Ca (700–1200 mg), Ca intake for people from 74 countries, especially from Asia, Africa, and South America, was lower than 700 mg/day [6]. Moreover, Ca deficiency is also common in older women and often causes other diseases.

Ca deficiency is often caused by insufficient Ca intake and poor bioavailability. However, dietary intake alone may not be sufficient to meet the Ca levels required by the body’s physiology. Therefore, adequate Ca intake and high bioavailability are profoundly important to maintain health benefits. Presently, various types of oral Ca supplements have been developed to resolve the problem of Ca deficiency, such as Ca gluconate, Ca lactate, and Ca carbonate [7]. Unfortunately, most Ca supplements have low bioavailability in vivo and poor effects in clinical practice due to their relatively low absorption rate, poor solubility, gastrointestinal irritation, and strong side effects [1].

In the past decades, protein hydrolysates and bioactive peptides (BPs) have been widely produced using different animal and plant proteins and engaged in human nutrition applications [8,9,10]. Upon proteolysis, the generated BPs with 2–20 amino acids have been regarded as high-quality nutritional supplements because of their low molecular weights (MW), resulting in easy absorption and high nutrition characteristics [11,12,13]. Moreover, BPs showed multifarious significant pharmacological functions, including antioxidant, antifatigue, antihypertensive, anti-aging, lipid-lowering, antimicrobial, and immunomodulatory activities [14,15,16]. In order to overcome the defects of Ca supplements in the current market, Ca-chelating peptides serving as a novel kind of Ca supplement have become the hot research theme [1]. Literature studies reported that Ca-chelating peptides could improve Ca absorbability and bioavailability by weakening the formation of insoluble Ca complexes and giving assistance to Ca transport into the blood. In addition, Ca-chelating peptides have the advantages of excellent stability and high safety [17,18]. Therefore, Ca-chelating peptides were produced using diverse animal and plant proteins, such as casein [19], tilapia [2], cattle bone [5], cucumber seed [4], mung bean [20], Pacific cod bone [21], oyster [22], Crimson snapper scales [18], and soy and pea [23].

Antarctic krill (*Euphausia superba*) is considered the world’s largest resource of animal proteins due to its huge biomass (342–356 million tons) and high biological value [24,25]. Therefore, Antarctic krill proteins are thought of as a high-quality resource for future marine healthy foods and functional products, and the studies on the preparation and activity of BPs from Antarctic krill proteins have gathered wide attention [26,27]. For example, phosphorylated peptides from Antarctic krill could ameliorate osteoporosis and alleviate liver fibrosis [25,28]; Antioxidant peptides, such as EYEA, SNVFDMF, QYPPMQY, AMVDAIAR, and LQP, could scavenge radical, protect liver cells, and liver organism against oxidative stress [29,30,31]; VLGYIQIR and LVDDHFL could be appropriate for novel mineral supplements [32,33]; SSDAFFPFR and SNVFDMF could ameliorate the memory impairment of mice induced by scopolamine [34]; WF, FAS, KVEPLP, and PAL presented strong ACE and/or DPP-IV inhibitory ability [35,36]. However, there are relatively few research studies on the preparation of metal-chelated peptides from Antarctic krill proteins. Therefore, the purposes of this work were to isolate and characterize the Ca-chelating peptides from the hydrolysate of Antarctic krill proteins. Furthermore, the chelating mechanism and absorption efficiency of prepared Ca-chelating peptides were studied using the Caco-2 cell monolayer model.

## 2. Results and Discussion

### 2.1. Preparation of Protein Hydrolysate of Antarctic Krill

#### 2.1.1. Screening of Protease Species

Antarctic krill proteins were hydrolyzed separately by five proteases, and the Ca-chelating rates of produced hydrolysates were presented in Table 1. The data indicated that the Ca-chelating rate of hydrolysate generated by trypsin was 37.91 ± 2.958%, which was remarkably greater than the rates of hydrolysates produced using flavourzyme (30.93 ± 1.37%), pepsin (24.11 ± 2.16%), papain (23.69 ± 1.98%), and alcalase (32.24 ± 2.31%), respectively (*p* < 0.05).

Enzymatic hydrolysis is the most applied process to generate Ca-chelating peptides on account of mild reaction conditions, high safety, and environmentally friendly features [1,36]. Therefore, some proteases, including prolyve enzyme [37], neutrase [3,22], protamex [38], alcalase [23], and bromelain [39], have been screened for the production of different mineral-chelating peptides. The present results further supported the opinion that the specificity of proteases remarkably influenced the Ca-chelating rates of hydrolysates. Therefore, the hydrolysate of Antarctic krill proteins produced using trypsin was prepared and named AKH.

#### 2.1.2. Optimized the Chelating Conditions of Ca with AKH

**Figure 1:**
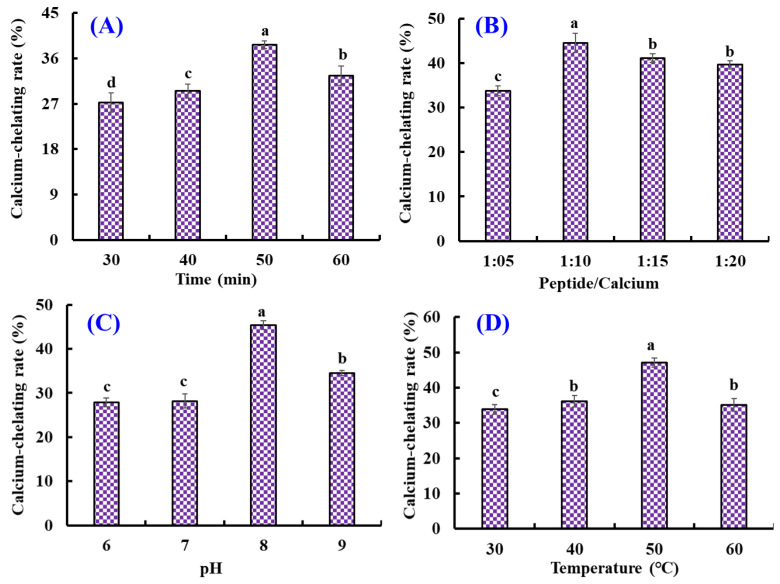
Effects of chelating time (**A**), peptide/Ca ratio (**B**), pH (**C**), and temperature (**D**) on the Ca-chelating rate of the hydrolysate of Antarctic krill proteins (AKH). ^a–d^ Values with the same letters indicate no significant difference (*p* > 0.05).

As shown in Figure 1, the effects of chelating conditions, including chelating time (30, 40, 50, and 60 min), temperature (30, 40, 50, and 60 °C), pH (6, 7, 8, and 9), and peptide/Ca ratio (1:5, 1:10, 1:15, and 1:20) on the Ca-chelating rate (%) of AKH were optimized by a single-factor experiment. Figure 1A illustrated that the Ca-chelating rate of AKH significantly (*p* < 0.05) increased when the chelating time increased from 30 to 50 min and achieved the highest value (38.68 ± 0.8%) at 50 min. Additionally, the Ca-chelating rate of AKH was markedly decreased when the chelating time ranged from 50 to 60 min. Figure 1B illustrated that the Ca-chelating rate of AKH significantly (*p* < 0.05) increased when the peptide/Ca ratio changed from 1:5 to 1:10 and achieved the highest value (44.57 ± 2.16%) at peptide/Ca ratio of 1:10. In addition, the Ca-chelating rate of AKH was markedly descent when the chelating time ranged from 50 to 60 min (*p* > 0.05). Figure 1C showed that the Ca-chelating rate of AKH was dramatically affected by the chelating pH, and the Ca-chelating rate (45.37 ± 0.96%) of AKH at pH 8.0 was remarkably higher than those of AKH at other pH (*p* < 0.05). Additionally, the Ca-chelating rate of AKH was a gradual decline when the pH value was higher than 8.0. Figure 1D indicated that chelating temperature significantly influenced the Ca-chelating rate of AKH, and the Ca-chelating rate (47.11 ± 1.31%) of AKH prepared at 50 °C was prominently stronger than that of hydrolysate prepared at other chelating temperatures (*p* < 0.05). Therefore, the range of chelating conditions for Ca with AKH was narrowed to chelating time (40, 50, and 60 min), temperature (40, 50, and 60 °C), pH (7, 8, and 9), and peptide/Ca ratio (1:5, 1:10, and 1:15), respectively.

Furthermore, the orthogonal test L_9_(3)^4^ was designed to optimize the chelating conditions for Ca with AKH (Table 2). Following the *R* values, the conditions interfering with the Ca-chelating rate of AKH were listed in decreasing order: A (chelating pH) > B (chelating time) > C (chelating temperature) > D (peptide/Ca ratio). The chelating pH was proved to be the most important condition influencing the Ca-chelating rate of AKH, and the optimal chelating level was A_2_B_2_C_3_D_3_; that is to say, the optimum chelating conditions of Ca with AKH were a chelating pH of 8.0, time of 50 min, temperature of 50 °C, and peptide/Ca ratio of 1:15.

### 2.2. Preparation of Ca-Chelating Peptides from AKH

#### 2.2.1. Ultrafiltration

**Figure 2:**
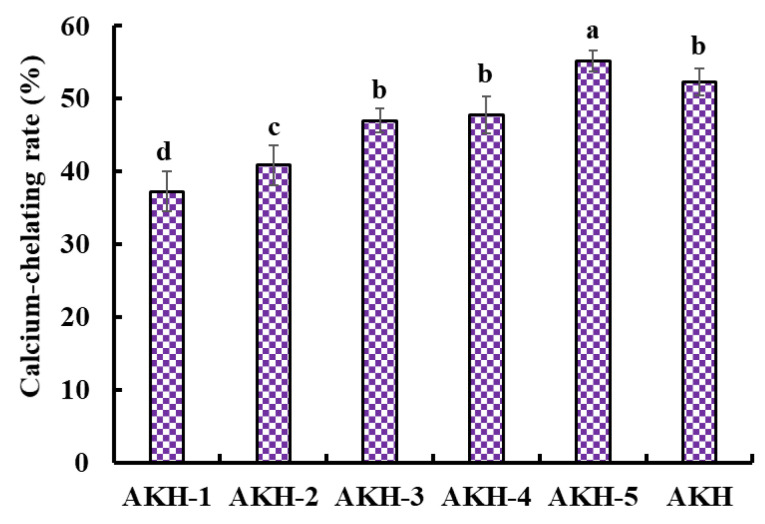
Ca-chelating rate of AKH and its fractions (AKH-1~AKH-5) by ultrafiltration. ^a–d^ Values with same letters indicate no significant difference (*p* > 0.05).

Dietary Ca forms Ca phosphate deposition in the gastrointestinal system, inducing poor absorbance [4]. However, peptides shape a peptide-Ca chelate with Ca^2+^, which increases the solubility and bioavailability of Ca in the intestine. The Ca absorption was influenced by the structure and Ca-chelating capability of peptides, so evaluating the Ca-chelating capability of different peptide components was necessary. In the experiment, five components, including AKH-1 (MW > 10 kDa), AKH-2 (5–10 kDa), AKH-3 (3.5–5 kDa), AKH-4 (1–3.5 kDa), and AKH-5 (MW < 1 kDa), were obtained from AKH. Figure 2 showed that the Ca-chelating rate of AKH-5 was observably higher than those of AKH and the other four fractions (*p* < 0.05). Ultrafiltration is a popular technique to concentrate target fractions from protein hydrolysates according to their molecular size [40,41]. After ultrafiltration, AKH-5 collected more low MW peptides, exposing more binding sites for Ca^2+^. Huang et al. reported that peptide fraction (<1 kDa) separated from the hydrolysate of shrimp byproducts exhibited greater affinity to Ca^2+^ [42]. In addition, low MW peptides are easier to digest and absorb in the body. Therefore, AKH-5 was chosen for further study.

#### 2.2.2. Anion-Exchange Chromatography of AKH-5

**Figure 3:**
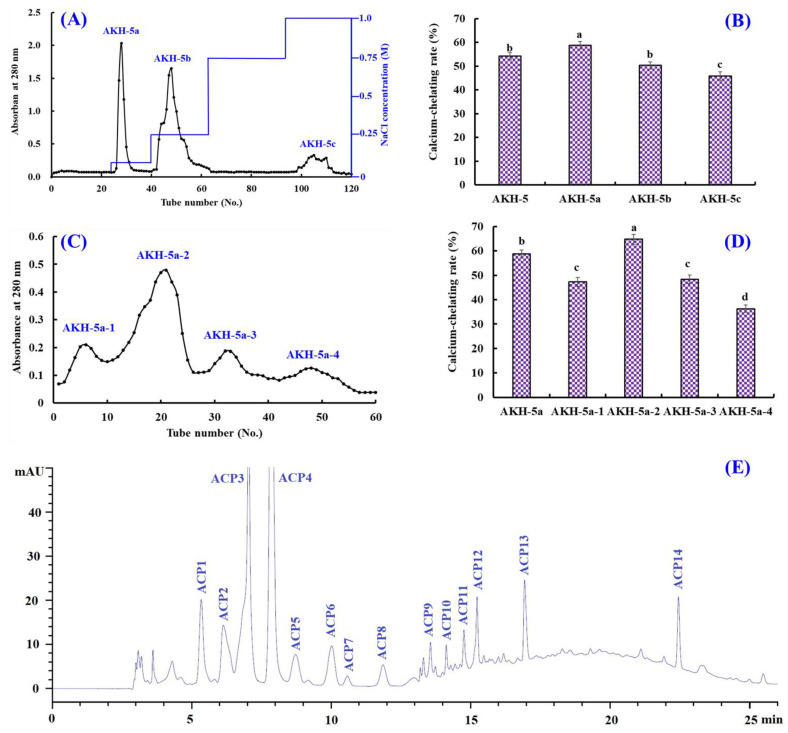
Isolation of Ca-chelating peptides from the ultrafiltration fraction AKH-5. (**A**) Elution profiles of AKH-5 in DEAE-52 cellulose chromatograph; (**B**) Ca-chelating ability of fractions from AKH-5. (**C**) elution profile of AKH-5a in Sephadex G-25 chromatography; (**D**) Ca-chelating ability of fractions from AKH-5a; and (**E**) elution profiles of AKH-5a-2 by RP-HPLC. ^a–d^ Values with same letters indicate no significant difference (*p* > 0.05).

Ion-exchange chromatography is popularly applied to separate polar molecules (such as proteins and peptides) from crude extraction and protolysate according to their affinities to ion exchangers. As shown in Figure 3A, AKH-5 was divided into three fractions (AKH-5a, AKH-5b, and AKH-5c) by a DEAE-52 cellulose column. The Ca-chelating rate of AKH-5a was 58.74 ± 1.64%, which was observably higher than those of AKH-5, AKH-5b, and AKH-5c (*p* < 0.05) (Figure 3B). Peptides usually have polar amino acid residues, which are easier to bind to the cation or anion exchange resins, such as XK 26 DEAE, SP-Sephadex C-25, DEAE-52 cellulose, AG 50W-X2, Q Sepharose FF, etc. [43]. Reddy and Mahoney [44] reported that peptides could exhibit stronger Ca-chelating activity if they contain more groups with more negative charges. In addition, Zhao et al. [45] found that other properties of peptides, such as MW and hydrophilicity/hydrophobicity, also have significant effects on the adsorption capacity between peptides and Ca. The Ca-chelating ability of AKH-5a should be the result of multiple effects, including negative charges, molecular size, and hydrophilicity/hydrophobicity. Therefore, AKH-5a was selected for further purification.

#### 2.2.3. Gel Permeation Chromatography (GPC) of AKH-5a

Molecular size is one of the key factors to consider in the purification of BPs [46,47]. Thus, AKH-5a was further divided into four fractions (AKH-5a-1, AKH-5a-2, AKH-5a-3, and AKH-5a-4) by a Sephadex G-25 column (Figure 3C). The Ca-chelating rate of AKH-5b-2 was 64.74 ± 1.98%, which was observably higher than those of AKH-5a, AKH-5a-1, AKH-5a-3, and AKH-5a-4, respectively (*p* < 0.05) (Figure 3D). Gel filtration is an efficient method to separate bioactive substances with different MW ranges and is widely applied for BP purification from different protein hydrolysates, such as whey [45], shrimp byproducts [42], Alaska pollock [48], monkfish [13], tilapia (*Oreochromis niloticus*) [49], *Mytilus edulis* [50], peanut [51], *Cyclina sinensis* [52,53], etc. Although AKH-5b-2 presented the best Ca-chelating ability, its MW was not the lowest among the four peptide fractions. These findings illustrated that other factors besides MW, such as amino acid composition and hydrophilicity/hydrophobicity, also greatly influence the Ca-chelating ability of peptides. Then, AKH-5a-2 was chosen for HPLC isolation.

#### 2.2.4. RP-HPLC Purification of AKH-5a-2

AKH-5a-2 was finally purified by RP-HPLC (Figure 3E). According to the elution profiles of AKH-5a-2 at 280 nm, fourteen Ca-chelating peptides with retention times (RTs) of 5.29 (ACP1), 6.18 (ACP2), 7.02 (ACP3), 7.95 (ACP4), 8.76 (ACP5), 10.01 (ACP6), 10.58 (ACP7), 11.91 (ACP8), 13.60 (ACP9), 14.11 (ACP10), 14.85 (ACP11), 15.22 (ACP12), 16.98 (ACP13), and 22.46 min (ACP14) were isolated and collected (Table 3). RP-HPLC is an extremely effective technology for purifying BPs according to their RT, and the RT of separated BPs can be modulated by adjusting the ratio of polar solvent (methanol and acetonitrile) in the mobile phase [50]. Therefore, RP-HPLC has been used to purify Ca-chelating peptides from protein hydrolysates of whey [45], casein [54], Alaska pollock [48], *Mytilus edulis* [50], phosvitin [55], miiuy croaker [46], tilapia (*Oreochromis niloticus*) [49], peanut [51], *Harpadon nehereus* [56], *Stolephorus chinensis* [57], etc.

### 2.3. Determination of Sequences and MWs of Ca-Chelating Peptides (ACP1–ACP14)

By Protein/Peptide Sequencer, the fourteen Ca-chelating peptide (ACP1–ACP14) sequences were identified as Ala-Lys (AK, ACP1), Glu-Ala-Arg (EAR, ACP2), Ala-Glu-Ala (AEA, ACP3), Val-Glu-Arg-Gly (VERG, ACP4), Val-Ala-ESr (VAS, ACP5), Gly-Pro-Lys (GPK, ACP6), Ser-Pro (SP, ACP7), Gly-Pro-Lys-Gly (GPKG, ACP8), Ala-Pro-Arg-Gly-His (APRGH, ACP9), Gly-Val-Pro-Gly (GVPG, ACP10), Leu-Glu-Pro-Gly-Pro (LEPGP, ACP11), Leu-Glu-Lys-Gly-Ala (LEKGA, ACP12), Phe-Pro-Pro-Gly-Arg (FPPGR, ACP13), and Gly-Glu-Pro-Gly (GEPG, ACP14), and their MWs were determined as 217.27, 374.40, 289.29, 459.50, 275.30, 300.36, 202.21, 357.41, 536.59, 328.37, 511.58, 516.60, 572.66, and 358.35 Da, respectively, which were in good agreement with their theoretical MWs (Table 3).

### 2.4. Ca-Chelating Ability of Fourteen Isolated Peptides (ACP1–ACP14)

**Figure 4:**
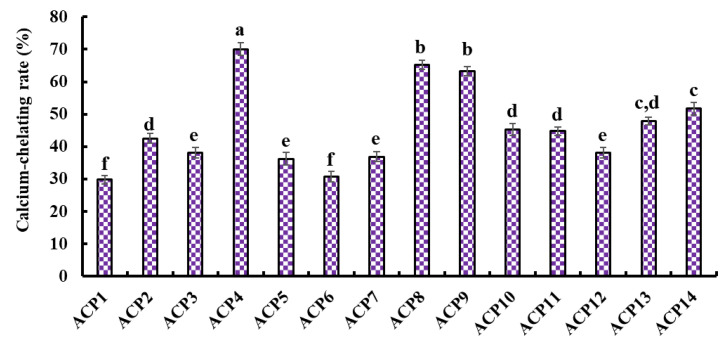
Ca-chelating ability of fourteen isolated peptides (ACP1-ACP14) by RP-HPLC. ^a–f^ Values with same letters indicate no significant difference (*p* > 0.05).

Figure 4 presents the Ca-chelating ability of fourteen isolated peptides (ACP1–ACP14). ACP4 (VERG) (70.05 ± 1.91%) showed the highest Ca-chelating ability among fourteen isolated peptides (ACP1–ACP14), and other peptides with higher Ca-chelating ability were followed by ACP8 (GPKG) and ACP9 (APRGH), respectively.

Amino acid composition and molecular size are two key factors affecting the Ca-chelating ability of peptides [19]. Peptides with short amino acid sequences, such as TCH, YDT, VLPVPQK, LLLGI, AIVIL, HADAD, YGTGL, LVFL, and LPEPV, were proved to contribute more to their Ca-chelating ability [19,41,44,48,50,52]. Guo et al. also reported that the Ca-chelating activity of tripeptides (SAC and SCH) was higher than those of SGSTGH, GPAGPR, and GPAGPHGPPG [48]. The MW of ACP4 (VERG) was 459.50 Da, which helped it easily interact with Ca^2+^ to form peptide-Ca chelate.

Amino acid composition is another widely recognized key factor that significantly influences the Ca-chelating ability of peptides. For example, Asp, Glu, and Gly residues were considered the major amino acid residues contributing to the Ca-chelating ability of peptides from porcine blood plasma [58]. Asp, Glu, Cys, and His residues are favorable for the metal-chelating activity of GPAGPHGPPG from Alaska pollock skin [48]. Liao et al. [54] indicated that Val, Pro, and Gln could significantly contribute to the high Ca-chelating ability of VLPVPQK. In general, the carboxyl group of Glu and Asp are favorable to bind Ca^2+^ because the carboxylic acid group can create an environment conducive to the chelating reaction of peptide and Ca by increasing the charge density [22]. The contents of hydrophobic amino acids, such as Val, Pro, and Leu, were also proved to be associated with the amount of Ca bound [59]. Gly residue could maintain the strong flexibility of the peptide skeleton to easily access and bind to Ca^2+^ [9]. In addition, Liu et al. [59] found that Arg was one of the main amino acid residues for the Ca-chelating peptides from wheat germ. Therefore, Val, Glu, Arg, and Gly residues should be very helpful in improving the Ca-chelating ability of ACP4 (VERG). Therefore, ACP4 (VERG) was selected to chelate with Ca and used for the next experiment.

### 2.5. Characterization of VERG-Ca Chelate

#### 2.5.1. UV Absorption Spectroscopy

**Figure 5:**
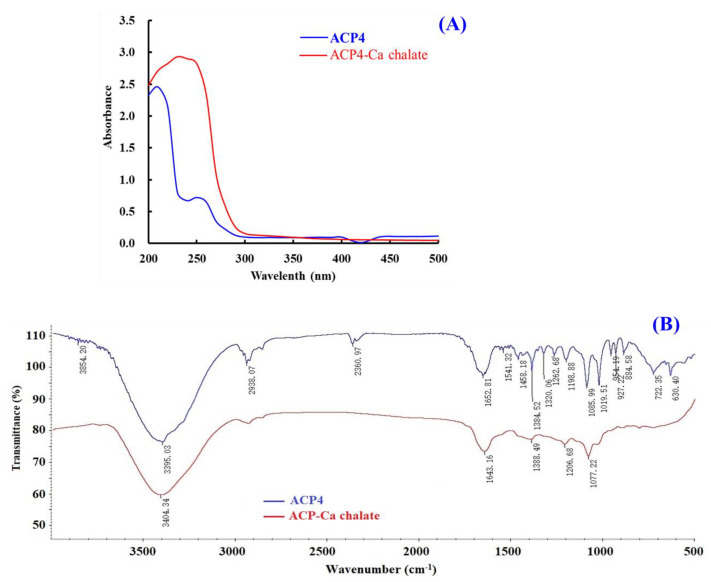
Characterizations of ACP4 (VERG) and VERG-Ca chelate. (**A**) UV spectra of ACP4 (VERG) and VERG-Ca chelate in the regions from 200 to 500 nm. (**B**) FTIR spectra of ACP4 (VERG) and VERG-Ca in the 4000 to 500 cm^−1^.

UV spectrum is a popular method to study the structural characteristics of substances and their derivatives. The appearance of new absorbance peaks or the changes in the pre-existing peaks in the UV spectrum indicate the formation of chelates consisting of organic ligands and metal ions [55]. Figure 5A indicated that the maximum absorption band of ACP4 (VERG) was found near 230 nm, caused by the n→π * transition of C=O in peptide bonds. The maximum absorption peak of the VERG-Ca chelate was 210 nm, suggesting that ACP4 binding with Ca^2+^ induced the absorption peak to shift towards the short wavelength. N and O in ACP4 (VERG) form a complex bond with Ca^2+^, which influences the electronic transition of C=O and –NH_2_ of the peptide bond. Our result is similar to the research results of peptide-Ca chelates from phosvitin [55], egg white [60], cucumber seed [4], whey [45], and sheep bone [61]. This present finding indicated that ACP4 (VERG) interacting with Ca^2+^ finally formed a new VERG-Ca chelate.

#### 2.5.2. FTIR Spectroscopy

FTIR spectroscopy is a crucial method for the research of the structural features of peptide-Ca chelates and can effectively reflect the mutual effect between peptides’ ligands and Ca^2+^ during the chelating process [51]. The peptides’ chelating sites with Ca^2+^ are primarily amide bonds (-CONH-) between amino acid residues and carboxyl (-COOH) and amino (-NH_2_) groups [55]. Then, the absorption peaks, such as stretching vibration of -NH_2_ and –COOH, are sure to shift if peptides chelate with Ca. The amide A band was attributed to the stretching vibration of the N-H bond. Figure 5B showed that the wavenumber moved from 3395.03 to 3404.34 cm^−1^ probably because of the stretching and substitution of hydrogen bonds in the VERG-Ca chelate, which manifested the participation of N–H bonds in the formation of chelate. The wavenumber (1700–1600 cm^−1^) of the amide-I band caused by C=O stretching vibration was moved from 1643.16 to 1652.81 cm^−1^, which indicates infrared absorption of the C=O caused by the antisymmetric stretching vibration of carboxylic acid ions. The wavenumber (1384.52 cm^−1^) for –COO– moved to a lower frequency (1388.49 cm^−1^) in the spectrum of the bound peptide and showed that –COOH probably bound Ca^2+^ and turned into –COO–Ca. These results were in line with the report of [55]. Based on these findings, we hypothesized that the N–H, C=O, and –COOH took responsibility for the chelation between ACP4 (VERG) and Ca^2+^.

#### 2.5.3. Scanning Electron Microscope (SEM)

**Figure 6:**
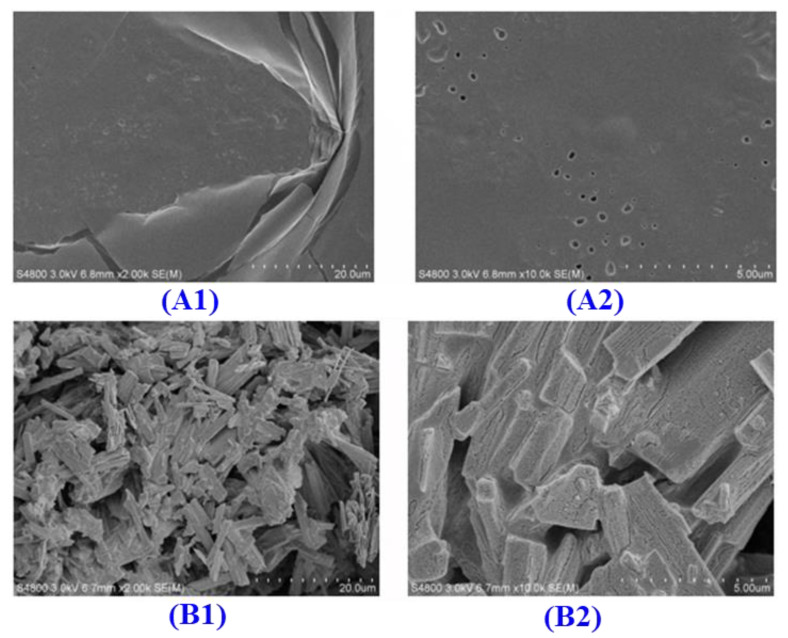
Scanning electron microscopy analysis of ACP4 (VERG) and VERG-Ca chelate. (**A1**) ACP4 (×500); (**A2**) (×2000); (**B1**) VERG-Ca (×500); and (**B2**) VERG-Ca (×2000).

The microstructures of ACP4 (A) and the VERG-Ca chelate (B) are displayed in Figure 6. The surface of ACP4 (VERG) is smooth. However, the surface of the VERG-Ca chelate was rougher and looser and had many irregular strips and granular aggregates. The significant difference in microstructure between ACP4 (VERG) and the VERG-Ca chelate might be caused by the interactions that ACP4 (VERG) reacted with Ca^2+^, leading to damage to the smooth structure of the ACP4 (VERG) surface. Additionally, –COOH and –NH_2_ in ACP4 (VERG) bound to Ca^2+^ and formed a “bridging role”, which could also change the physicochemical characteristics of ACP4 (VERG) [51].

### 2.6. Stability Analysis of VERG-Ca Chelate

**Figure 7:**
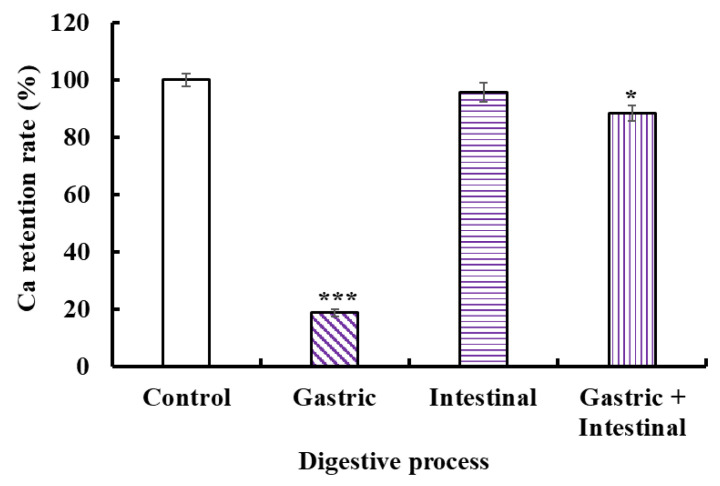
Stability of VERG-Ca chelate in simulated digestive tract environment. * *p* < 0.05 and *** *p* < 0.001 vs. Control.

The acid environment and protease in the gastrointestinal system could cause Ca release to generate Ca(OH)_2_ and insoluble precipitation, leading to low bioavailability of Ca. Therefore, we studied the stability of the VERG-Ca chelate in a simulated digestive tract environment (Figure 7). Compared with the control, the Ca-retention rates of the VERG-Ca chelate in gastric juice, intestinal juice, and gastric+intestinal juice decrease to 18.72 ± 1.25% (*p* < 0.001), 95.67 ± 3.26% (*p* > 0.05), and 87.21 ± 2.73% (*p* < 0.05), respectively. In addition, the Ca-retention rate of the VERG-Ca chelate in intestinal juice is significantly higher than that of gastric juice (*p* < 0.001). The finding indicated that the gastric environment significantly reduced the stability of the VERG-Ca chelate. It is worth noting that the Ca-retention rate of the VERG-Ca chelate in gastric+intestinal juice is significantly higher than that of gastric juice (*p* < 0.001) but was not much different from that of the intestinal group. The result indicated that pepsin could lead to the partial degradation of the chelate, except for the effect of pH. Cui et al. [62] and Zhang et al. [31] reported that the main reason for Ca^2+^ release in the gastrointestinal system was pH change, and the weak alkaline environment of the intestines could induce the rechelation of peptides with Ca^2+^. Our present finding in the gastric + intestinal juice group was in agreement with these literature studies. Therefore, gastric digestion is the key reason causing Ca to be released from the VERG-Ca chelate, but intestinal digestion had no remarkable effect on the stability of the VERG-Ca chelate. In general, most VERG-Ca chelates can remain stable in gastrointestinal digestion, which might prominently improve the bioavailability of Ca in the digestive tract environment.

### 2.7. Transport of VERG-Ca Chelate across the Caco-2 Cell Monolayer

**Figure 8:**
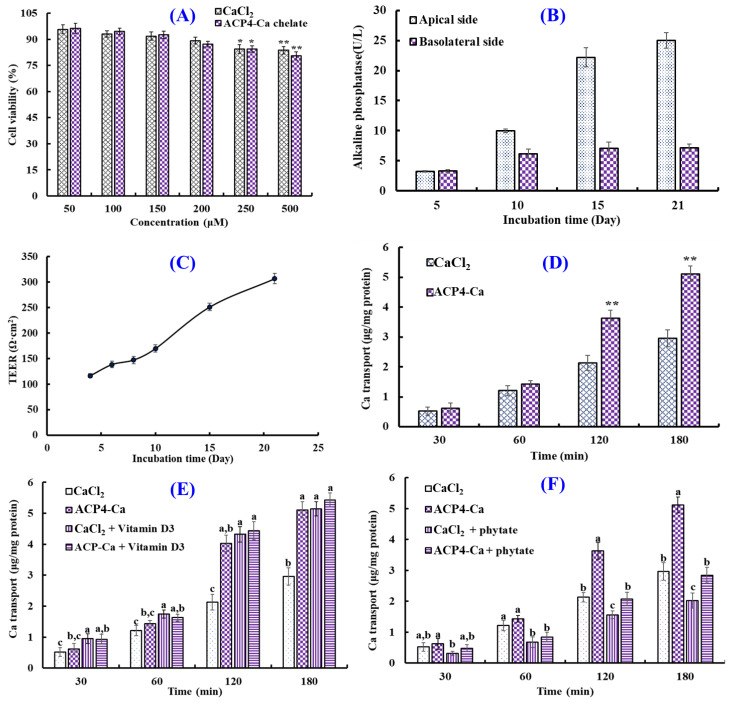
Transport of VERG-Ca chelate. (**A**) Cytotoxicity of VERG-Ca chelate in Caco-2 cells evaluated by the MTT assay. * *p* < 0.0 and ** *p* < 0.01 vs. control group. (**B**) Alkaline phosphatase activities on the apical and basolateral sides of the Caco-2 cell monolayer. (**C**) Transepithelial electrical resistance (TEER) of the Caco-2 cell monolayer. (**D**) Ca transport activity of VERG-Ca chelate. ** *p* < 0.01 vs. CaCl_2_ group at same concentration. (**E**) Effects of vitamin D3 on the transmembrane transport of CaCl_2_ and VERG-Ca chelate. (**F**) Effects of phytate on the transmembrane transport of CaCl_2_ and VERG-Ca chelate. ^a–c^ Values with same letters at same time indicate no significant difference (*p* > 0.05).

Human intestinal epithelial cells modeled in vitro using Caco-2 cells have been applied to study the simulative absorption of minerals, medicines, and peptides [46]. In this study, the effects of the VERG-Ca chelate on the viability of Caco-2 cells were measured by the MTT assay (Figure 8A). Compared with the control group, ACP4 (VERG) and the VERG-Ca chelate had no significant effect on cell viability at 50–200 μM, but they could significantly decrease the viability of Caco-2 cells at 250 μM (*p* < 0.05). The finding manifested that ACP4 (VERG) and the VERG-Ca chelate were nontoxic at concentrations of 50–200 μM. Therefore, the following experiment can be carried out in the concentration range of 0–200 μM.

Figure 8B shows the activity changes in alkaline phosphatase of Caco-2 cells’ monolayer membrane at 0–21 days. When Caco-2 cells were cultured to day 21, the activity of alkaline phosphatase on the apical side was 25.02 ± 1.32 U/L, which was 3.48-fold of that on the basolateral side (7.18 ± 0.63 U/L), indicating that Caco-2 cells completed the polar differentiation of the monolayer membrane. In addition, the change in TEER values of the Caco-2 cell monolayer within incubation time (1–21 days) was measured to create monolayers of Caco-2 cells with enterocyte architecture (Figure 8C). The results indicated that the TEER value gradually increased with the extension of incubation time, and the TEER exceeded 300 Ω cm^2^ at 21 days. Therefore, the Caco-2 cell monolayer model was applied to evaluate the influence of the VERG-Ca chelate on Ca transport activity.

The effect of the VERG-Ca chelate on Ca transport in Caco-2 cell monolayers is presented in Figure 8D. The Ca transport of the VERG-Ca chelate increased with the incubation time. Compared with the CaCl_2_ group, VERG-Ca chelate-treated groups did not display remarkable Ca transport capability in Caco-2 cell monolayers from 30 to 60 min (*p* < 0.05). However, the Ca transport improved dramatically in the presence of the VERG-Ca chelate at 120 and 180 min, and the quantity of Ca of transport reached 3.63 ± 0.27 and 5.11 ± 0.26 μg/mg protein at 120 and 180 min, which were 1.70 and 1.72 times of the CaCl_2_ group, respectively. Previous literature studies indicated that Ca-chelating peptides, including VLPVPQK [54], EYG [42], and FPPDVA [51], could greatly improve the Ca transport across Caco-2 cell monolayers. The present findings demonstrated that the Ca transport and absorption in Caco-2 cell monolayers could be increased by the VERG-Ca chelate.

Plant-based diets are rich in bioactive compounds, but some compounds, such as vitamins, tannins, phytates, and dietary fibers (DFs), can significantly influence the bioavailability of some minerals, especially Ca [63]. Therefore, the effects of vitamin D and phytate on the absorption of Ca derived from CaCl_2_ and VERG-Ca chelates were evaluated. Figure 8E indicated that the absorption amount of Ca significantly increased from 0 to 180 min after adding CaCl_2_ and vitamin D3 together, and the absorption amount of Ca increased from 2.96 ± 0.28 to 5.14 ± 0.23 μg/mg protein at 180 min, indicating that vitamin D3 can promote Ca uptake by regulating the transcellular pathway of Ca^2+^. In addition, the absorption amount of Ca significantly increased from 4.03 ± 0.27 to 4.44 ± 0.29 μg/mg protein at 120 min after adding VERG-Ca chelates and vitamin D3 together, and the absorption amount of Ca only increased from 5.11 ± 0.36 to 5.43 ± 0.43 μg/mg protein at 180 min, and no significant difference was observed. Therefore, it could be speculated that the vitamin D3 in food has no significant effect on intestinal absorption of Ca derived from VERG-Ca chelates but can significantly promote the absorption of inorganic Ca.

Figure 8F showed that the absorption amount of Ca decreased significantly at 0~180 min after adding phytate with CaCl_2_ or a VERG-Ca chelate. At 180 min, the absorption amount of Ca derived from CaCl_2_ decreased from 2.96 ± 0.28 to 2.02 ± 0.24 μg/mg protein. The absorption amount of Ca derived from the VERG-Ca chelate decreased from 3.63 ± 0.35% to 2.842 ± 0.348 μg/mg protein. The data indicated that phytate could significantly reduce the absorption amount of Ca derived from CaCl_2_ or the VERG-Ca chelate in Caco-2 cells, which was in agreement with the report by Amalraj and Pius [63] that Ca bioavailability in green leafy vegetables was negatively correlated with those anti-nutritional factors, such as dietary fiber, phytate, oxalate, and tannin.

## 3. Materials and Methods

### 3.1. Chemicals and Reagents

Antarctic krill was provided by Zhejiang Hailisheng Biotechnology Co., Ltd. (Zhoushan, China). Triethanolamine, trypsin, and Hanks’ Balanced Salt Solution (HBSS) were purchased from Sigma-Aldrich (Shanghai) Co., Ltd. (Shanghai, China). Methanol, potassium hydroxide, hydrochloric acid, sodium chloride, ferrous sulfate, calcium indicator, and sodium cyanide were purchased from Sinopharm Chemical Reagent Co., Ltd. (Shanghai, China). The peptides (ACP1-ACP14) with purity higher than 98% were synthesized by Shanghai Apeptide Co., Ltd. (Shanghai, China).

### 3.2. Preparation of Protein Hydrolysate of Antarctic Krill

#### 3.2.1. Screening of Protease Species

The degreasing process of Antarctic krill was performed using the described method by Zhao et al. [46]. Defatted Antarctic krill powders were dispersed in distilled water (DW) (10%, *w*/*v*) and separately hydrolyzed using different proteases (Table 1). After that, the hydrolysis reaction was stopped at 95 °C for 15 min and centrifuged at 9000× *g* at −4 °C for 20 min. The resulting supernatants were lyophilized and stored at −20 °C. The hydrolysate prepared by trypsin displayed the strongest Ca-chelating rate and was named AKH.

#### 3.2.2. Optimization of the Chelating Conditions of Ca with AKH

Firstly, the chelating conditions of Ca with AKH were optimized by a single-factor experiment using Ca-chelating rate as the indicator. The chelating time (30, 40, 50, and 60 min), temperature (30, 40, 50, and 60 °C), pH (6, 7, 8, and 9), and peptide/Ca ratio (1:5, 1:10, 1:15 and 1:20) were chosen for the present investigation.

According to the results of single-factor experiment, orthogonal experiment was employed to estimate the influence of chelating time (40, 50, and 60 min), temperature (40, 50, and 60 °C), pH (7, 8, and 9), and peptide/Ca ratio (1:5, 1:10 and 1:15) on the Ca-chelating rate of AKH.

#### 3.2.3. Preparation of Peptide-Ca Chelates and Determination of Ca-Chelating Rate

Peptide-Ca chelates were prepared in accordance with the previous method [5]. The lyophilized AKH, AKH fractions, and peptides were separately dissolved in DW, and CaCl_2_ was added to the prepared solution (50 mg/mL) with designed peptide/CaCl_2_ ratios of 1:5–1:25 (*w*/*w*). The pH of the reaction mixtures was adjusted to pH 8.0. Then, the solution was put in a water bath shaker to incubate at 50 °C and 80 r/min for 50 min. Finally, the chelates were precipitated for 4 h by adding 5 times the volume of the solution of anhydrous ethanol. After that, the solution was centrifuged at 6000× *g* for 15 min, and the precipitation was collected, lyophilized, and stored at −20 °C. The Ca content in the supernatant was measured by EDTA complexion titration (ECT) [59], which was denoted as C2 (g/mL). The total Ca content in mixed solution was measured by ECT assay, which was denoted as C1 (g/mL). The Ca-chelating rate was calculated as follows:

Ca-chelating rate (%) = (C1 − C2/C1) × 100%.

#### 3.2.4. Separation Process of Ca-Chelating Peptides from AKH

AKH was ultra-filtrated by 4 kinds of ultrafiltration membranes, including 1, 3.5, 5, and 10 kDa. Then, five peptide components, including AKH-1 (MW > 10 kDa), AKH-2 (5–10 kDa), AKH-3 (3.5–5 kDa), AKH-4 (1–3.5 kDa), and AKH-5 (MW < 1 kDa), were collected, concentrated, and freeze-dried, and their Ca-chelating rates were measured using the above method in Section 3.2.3.

AKH-5 (10 mL, 50.0 mg/mL) was added into a pre-equilibrated DEAE-52 cellulose column (3.8 × 150 cm) and eluted by DW, 0.10, 0.25, 0.50, 0.75, and 1.0 M NaCl solution, respectively. The flow rate of eluate was set as 3.0 mL/min. Then, three fractions (AKH-5a, AKH-5b, and AKH-5c) were separated according to the DEAE-52 cellulose chromatography of eluted peptide fractions (9 mL) at 280 nm.

AKH-5a (5 mL, 50.0 mg/mL) was loaded into a Sephadex G-25 column (2.6 cm × 120 cm) and eluted using DW. The flow rate of eluate was set as 0.6 mL/min, and the eluent was collected every 3 min. Finally, four fractions (AKH-5a-1 to AKH-5a-4) were isolated from AKH-5a and collected in accordance with the chromatographic peaks at 280 nm.

**Figure 9:**
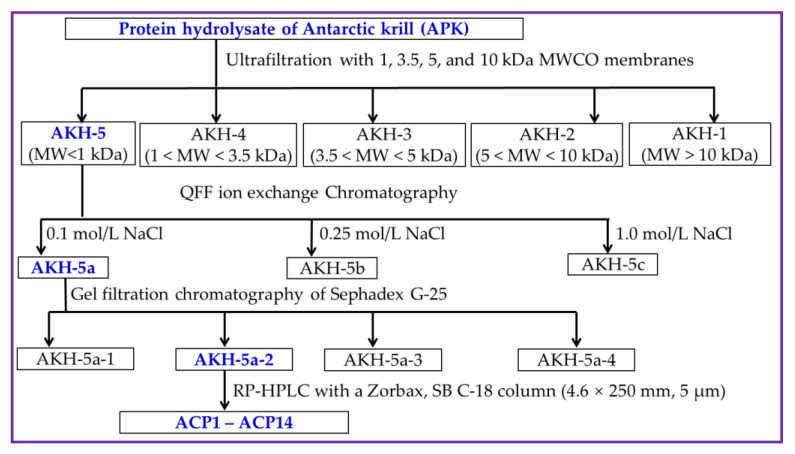
The flow diagram of separation process of Ca-chelating peptides from AKH.

AKH-5a-2 solution was decontaminated through a 0.22 μM microporous membrane and further isolated by an RP-HPLC column of Zorbax, SB C-18 (4.6 × 250 mm, 5 µm). In brief, AKH-5a-2 was loaded into the RP-HPLC column and eluted by a linear gradient of acetonitrile with a flow rate of 1.0 mL/min. The concentration ranged from 0 to 50% in 0 to 25 min, and the eluent was monitored at 280 nm. Finally, fourteen Ca-chelating peptides (ACP1 to ACP14) were prepared according to their chromatographic peaks. The flow diagram of separation process of Ca-chelating peptides from AKH is presented in Figure 9.

### 3.3. Identification of Peptides (ACP1to ACP14) from AKH

The amino acid sequence and molecular weight of EP1-EP6 were determined according to previous methods described by Chi et al. [64]. The amino acid sequences of ACP1 to ACP14 were measured by a 494 protein sequencer from Applied Biosystems (Perkin Elmer Co. Ltd. Foster City, CA, USA), and Edman degradation was performed according to the standard program supplied by Applied Biosystems. The MWs of ACP1 to ACP14 were determined by a Q-TOF mass spectrometer with an ESI source (Micromass, Waters, Milford, MA, USA).

### 3.4. Characterization of VERG-Ca Chelate

The preparation of a hydrolysate of PSC-M was carried out in accordance with the previous method [34,42]. The dispersions (1%, *w*/*v*) of ASC-M and PSC-M were separately degraded with alcalase (55 °C, pH 8.5, 4 h), neutrase (55 °C, pH 7.0, 4 H), and a double-enzyme system (alcalase (2 h) + neutrase (2 h)). The enzyme dose was designed as 2% (*w*/*w*). After the hydrolysis reaction, the proteases in the hydrolysate solution were inactivated in boiling water for 10 min. The prepared hydrolysates were centrifuged at 9000× *g* for 25 min, and the supernatants were freeze-dried and had their ACEi abilities detected. The hydrolysate of PSC-M generated via the double-enzyme system displayed the highest ACEi ability value and was named PSC-MH.

#### 3.4.1. UV Absorption Spectroscopy Analysis

The UV spectra of ACP4 and VERG-Ca chelate at 1.0 mg/mL were recorded in the wavelength range of 200–500 nm by a UV-1800 spectrophotometer (Mapada Instruments Co., Ltd., Shanghai, China).

#### 3.4.2. FTIR Spectroscopy Analysis

The FTIR spectra (4000 to 400 cm^−1^) of ACP4 and VERG-Ca chelate were recorded in KBr disks using a Nicolet 6700 FTIR spectrophotometer [65]. Dry ACP4 or VERG-Ca chelate was uniformly ground with dry KBr at a sample/KBr ratio of 1:100 (*w*/*w*) and extruded into a transparent sheet for spectrum recording.

#### 3.4.3. Scanning Electron Microscopy (SEM) Analysis

A suitable amount of ACP4 and VERG-Ca chelate was uniformly applied to the sample plate and sprayed with a gold plating film. Finally, the samples with a gold plating film were observed and photographed by a scanning electron microscope of JEOL JSM-6390LV (Tokyo, Japan).

### 3.5. Stability Analysis of VERG-Ca Chelate against Simulated Gastrointestinal Digestion

The stability of VERG-Ca chelate against simulated gastrointestinal digestion was assessed using the method described by Zhang et al. [31]. The VERG-Ca chelate solution (10 mg/mL) dissolved in DW was used in the assay. The pH values of the artificial simulation of gastric juice and intestinal juice were adjusted to 2.0 and 7.5, respectively. The stability of VERG-Ca chelate against in vitro gastric juice (2 h), intestinal juice (2 h), and gastric juice (2 h) + intestinal juice (2 h) were determined at 37 °C, respectively. Finally, the solution was heated at 100 °C for 15 min to terminate the reaction. The stability was expressed as Ca-retention rate of VERG-Ca chelate after in vitro digestion.

Ca-retention rate (%) = (Ca content in treatment group/Ca content in control group) × 100

### 3.6. Ca Transport Effect of VERG-Ca Chelate in Caco-2 Cell Monolayers

#### 3.6.1. Culture of Caco-2 Cells and Establishment of Caco-2 Cell Monolayer Model

Caco-2 cells were obtained from the Shanghai Institute of Cell Biology (Shanghai, China) and cultured in EMEM with 10% fetal bovine serum, 1% antibiotic, and 1% nonessential amino acids in a humidified incubator at 37 °C with 5% CO_2_. The cell culture medium was replaced every two days with a new medium of equal volume. The Caco-2 cells were dispersed using trypsin-EDTA when they covered approximately 90% of the flask and were seeded on 6-well transwell culture plates with polyester membranes. The medium was replaced every other day with a new medium of equal volume. After being cultured for 21 days, the transepithelial electrical resistance (TEER) was determined to confirm the integrity of the Caco-2 cell monolayers. The Caco-2 cell monolayers were used for Ca transport experiments when the TEER exceeded 300 Ω·cm^2^ [60,66].

In addition, the alkaline phosphatase activity was determined using assay kits in accordance with the instructions of Nanjing Jiancheng Bioengineering Institute (Nanjing, China).

#### 3.6.2. Cytotoxicity Assay

Cell cytotoxicity was determined using the MTT assay [60]. After being cultured for 24 h, the Caco-2 cells were further treated for 24 h using a 100 μL medium containing VERG-Ca chelate with concentrations of 50, 100, 150, 200, and 500 μM, respectively. Then, MTT solution (100 μL) was added per well and incubated for an additional 4 h. The MTT solution per well was replaced by an equal volume of DMSO solution to solubilize. Finally, the absorbance of the sample solution at 570 nm was measured.

#### 3.6.3. Ca Transport Analysis

The Ca transport in the Caco-2 cell monolayer model was analyzed using previous methods [51,60]. After 21 days of incubation, the culture medium of Caco-2 cells was discarded from each well, and monolayers were immediately washed with HBSS buffer (without Ca and magnesium) two times. The Caco-2 cell monolayers were transferred to a new 6-well culture plate containing 2 mL of HBSS buffer, and 2 mL of HBSS buffer was also added to the apical side. After incubating for 30 min, the HBSS on the apical side was replaced with 2 mL of HBSS containing VERG-Ca chelate (200 μM) or Ca (150 μg/mL), and the HBSS on the basolateral side was replaced with an equal volume of fresh HBSS buffer. After incubation for 2 h, 1 mL of HBSS in the basolateral side was removed to determine Ca content at the designed time (30, 60,120, and 180 min). At the same time, fresh HBSS buffer (1 mL) was added to the basolateral side to maintain a constant volume. The content of Ca was determined by atomic absorption spectrometry using HNO_3_ and HClO_4_ as the oxidizers.

In the experiments on the effects of vitamin D3 and phytate on Ca absorption, dietary factors (vitamin D3 and phytate) were first premixed with VERG-Ca chelate or CaCl_2_, respectively, at a mass ratio of 1:1. The experiment conditions and operation method were as described in the Ca transport analysis above.

### 3.7. Statistical Analysis

The experimental data were represented by the mean ± standard deviation (SD, *n* = 3). GraphPad Prism (version 8.02) was used for one-way analysis of variance, and Tukey’s multiple comparison tests were used to test for differences between groups (*p* < 0.05).

## 4. Conclusions

In brief, fourteen Ca-chelating peptides were purified and identified from the trypsin hydrolysate of Antarctic krill proteins, and ACP4 (VERG) presented the highest Ca-chelating ability among isolated Ca-chelating peptides. UV, FTIR, and SEM analysis showed N-H, C=O, and -COOH in ACP4 (VERG) should take responsibility for the chelation of ACP4 (VERG) with Ca^2+^. Moreover, VERG-Ca chelate is stable in gastrointestinal digestion and could significantly improve Ca transport in Caco-2 cell monolayer experiments, but phytate could significantly reduce the absorption of Ca derived from VERG-Ca chelate. The present results suggested that Ca-chelating peptides derived from Antarctic krill proteins could serve as functional ingredients in healthy food to promote Ca bioavailability. However, the absorption and transport mechanisms of VERG-Ca chelate in vivo need to be further elucidated.

## Figures and Tables

**Table 1:** Effects of different proteases on Ca-chelating rate (%) of protein hydrolysates from Antarctic krill.

Protease	Enzymolysis Condition				Ca-Chelating Rate (%)
	Temperature (°C)	Time (h)	Enzyme Dose (%)	pH	
FlavourzymeAPePepsin	50	4	2.0	7.0	30.93 ± 1.37 ^b^
	37.5	4	2.0	2.0	24.11 ± 2.16 ^c^
Trypsin	37.5	4	2.0	7.8	37.91 ± 2.96 ^a^
Papain	55	4	2.0	7.0	23.69 ± 1.98 ^c^
Alcalase	55	4	2.0	9.5	32.24 ± 2.31 ^b^

^a–c^ Values with different letters indicate significant difference (*p* < 0.05).

**Table 2:** Results of the L_9_(3)^4^ orthogonal experiment for optimizing the chelating conditions of Ca with AKH.

No.	pH	Time (min)	Temperature (°C)	Peptide/Ca Ratio	Ca-Chelating Rate (%)
1	7	40	40	1:5	42.38
2	7	50	50	1:10	47.11
3	7	60	60	1:15	46.29
4	8	40	50	1:15	51.95
5	8	50	60	1:5	53.71
6	8	60	40	1:10	49.17
7	9	40	60	1:10	47.35
8	9	50	40	1:15	49.15
9	9	60	50	1:5	47.86
K1	135.78	141.68	140.7	143.95	
K2	154.83	149.97	146.92	143.63	
K3	144.36	143.32	147.35	147.39	
Best level	A2	B2	C3	D3	
*R*	19.05	8.29	6.65	3.72	
R order	A > B > C > D				

**Table 3:** Retention time, amino acid sequence, and molecular weight of 14 peptides (ACP1–ACP14) from AKH-5b-2.

No.	Retention Time (min)	Amino Acid Sequence	Observed/Theoretical MW (Da)
ACP1	5.29	Ala-Lys (AK)	217.27/217.27
ACP2	6.18	Glu-Ala-Arg (EAR)	374.40/374.39
ACP3	7.02	Ala-Glu-Ala (AEA)	289.29/289.29
ACP4	7.95	Val-Glu-Arg-Gly (VERG)	459.50/459.50
ACP5	8.76	Val-Ala-Ser (VAS)	275.30/275.30
ACP6	10.01	Gly-Pro-Lys (GPK)	300.36/300.35
ACP7	10.58	Ser-Pro (SP)	202.21/202.21
ACP8	11.91	Gly-Pro-Lys-Gly (GPKG)	357.41/357.41
ACP9	13.60	Ala-Pro-Arg-Gly-His (APRGH)	536.59/536.58
ACP10	14.11	Gly-Val-Pro-Gly (GVPG)	328.37/328.36
ACP11	14.85	Leu-Glu-Pro-Gly-Pro (LEPGP)	511.58/511.57
ACP12	15.22	Leu-Glu-Lys-Gly-Ala (LEKGA)	516.60/516.59
ACP13	16.98	Phe-Pro-Pro-Gly-Arg (FPPGR)	572.66/572.66
ACP14	22.46	Gly-Glu-Pro-Gly (GEPG)	358.35/358.35

## Data Availability

Data are contained within the article.
